# The Early Metazoan *Trichoplax adhaerens* Possesses a Functional *O*-GlcNAc System*[Fn FN2]

**DOI:** 10.1074/jbc.M114.628750

**Published:** 2015-03-16

**Authors:** Nithya Selvan, Daniel Mariappa, Henk W. P. van den Toorn, Albert J. R. Heck, Andrew T. Ferenbach, Daan M. F. van Aalten

**Affiliations:** From the ‡ Division of Molecular Microbiology and; § MRC Protein Phosphorylation and Ubiquitylation Unit, College of Life Sciences, University of Dundee, Dow Street, Dundee, DD1 5EH, United Kingdom and; the ¶ Biomolecular Mass Spectrometry and Proteomics, Bijvoet Center for Biomolecular Research and Utrecht Institute for Pharmaceutical Sciences, Utrecht University, Padualaan 8, 3584 CH Utrecht, The Netherlands

**Keywords:** Animal Model, Drosophila Genetics, Evolution, O-GlcNAcylation, Post-translational Modification (PTM), Trichoplax adhaerens

## Abstract

Protein *O*-GlcNAcylation is a reversible post-translational signaling modification of nucleocytoplasmic proteins that is essential for embryonic development in bilateria. In a search for a reductionist model to study *O*-GlcNAc signaling, we discovered the presence of functional *O*-GlcNAc transferase (OGT), *O*-GlcNAcase (OGA), and nucleocytoplasmic protein *O*-GlcNAcylation in the most basal extant animal, the placozoan *Trichoplax adhaerens*. We show via enzymatic characterization of *Trichoplax* OGT/OGA and genetic rescue experiments in *Drosophila melanogaster* that these proteins possess activities/functions similar to their bilaterian counterparts. The acquisition of *O*-GlcNAc signaling by metazoa may have facilitated the rapid and complex signaling mechanisms required for the evolution of multicellular organisms.

## Teaser

**Background:** Protein *O*-GlcNAcylation and orthologues of *O*-GlcNAc transferase (OGT) and *O*-GlcNAcase (OGA) occur separately or together in all kingdoms of life.

**Results:** The basal metazoan *Trichoplax adhaerens* is the simplest organism to possess functional OGT, OGA, and protein *O*-GlcNAcylation together.

**Conclusion:** Reversible protein *O*-GlcNAcylation is conserved throughout the metazoan lineage.

**Significance:** *T. adhaerens* can be used as a reductionist model to identify evolutionarily conserved *O*-GlcNAc targets.

## Introduction

Post-translational protein *O*-GlcNAcylation is the reversible addition of β-d-*N*-acetylglucosamine (GlcNAc) to serine and threonine residues on metazoan nucleocytoplasmic proteins (1). *O*-GlcNAc transferase (OGT)^2^ and *O*-GlcNAcase (OGA) are the enzymes responsible for the addition and removal of *O*-GlcNAc, respectively. Since it was first described in 1984 (2), *O*-GlcNAc has become associated with a range of cellular processes (1). There appears to be extensive cross-talk between *O*-GlcNAc and Ser/Thr phosphorylation, with the two modifications occurring at the same or neighboring residues on proteins (3–5). *O*-GlcNAcylation of several kinases (AMP-activated protein kinase, CaMKIV, and CaMKII, for example) regulates their activity, and OGT functionally interacts with two catalytic subunits of protein phosphatase 1 (6–9). The discovery of *O*-GlcNAc on proteasome subunits in *Drosophila* implicates a role for this post-translational modification in protein trafficking and degradation (10). *O*-GlcNAcylation increases in the presence of stressors like heat and heavy metals (11, 12), and protects cardiac tissues following ischemia (13). Reports have emerged of the involvement of *O*-GlcNAc in gene expression and epigenetics. The discovery of *O*-GlcNAc on RNA polymerase II transcription factors suggested a role for the modification in transcriptional activation (14). Overexpressing OGT in mitotic cells was shown to alter methylation and phosphorylation of histone H3 (15). In addition, cell cycle-dependent *O*-GlcNAc cycling was also found to occur on histones H2A, H2B, and H4 (16). Transcriptional repression by OGT involving interactions with mSin3A and HDAC1 has been demonstrated (17). Activation of gene expression downstream of H2B *O*-GlcNAcylation has been characterized, as well as transcriptional changes due to H3K4 trimethylation facilitated by the TET protein-OGT complex (18, 19). *O*-GlcNAcylation is associated with disease conditions like Type II diabetes, Alzheimer disease, and cancer (1, 20).

Following the identification of OGT and OGA activities (21–23) and their enzymatic characterization (23, 24), transcripts have been cloned from humans and other organisms (25, 26) and found to be highly conserved in animals. In humans, a single *OGT* gene encodes three isoforms of the protein, the longest, nucleocytoplasmic OGT (ncOGT/hOGT), is a ∼116 kDa protein and possesses 13.5 tetratricopeptide repeats (TPRs) at its N terminus (27, 28). Another isoform possessing 9.5 TPRs and a mitochondrial localization signal (mOGT ∼103 kDa) is targeted to mitochondria. The shortest OGT isoform (sOGT ∼78 kDa) contains only 2.5 TPRs and also has nucleocytoplasmic localization (27). In *Drosophila* and *Caenorhabditis elegans*, single *ogt* genes encode a single protein similar to human ncOGT (27, 29–31). Zebrafish is exceptional among animals to possess two *ogt* genes encoding six variants of the protein at different stages of development (32).

In humans, a single gene encodes two isoforms of OGA. The longer cytoplasmic isoform (hOGA ∼ 130 kDa) possesses an N-terminal catalytic domain and a C-terminal histone acetyltransferase (HAT)-like domain, whereas the shorter nuclear and lipid-droplet targeted isoform (∼75 kDa) lacks the HAT-like domain (33, 34). In *C. elegans*, a single *oga* gene encodes four major transcripts generated by alternative splicing and in-frame intron utilization to produce proteins of different lengths containing both the catalytic and HAT-like domains (35). *Drosophila* has a single *oga* gene encoding a single protein. Toleman *et al.* (36) demonstrated HAT activity for hOGA purified from mammalian cells, which was, however, not observed in a subsequent study (37). Structural characterizations of putative bacterial acetyltransferases sharing sequence conservation with the HAT-like domain of hOGA enforce that hOGA lacks HAT activity (38, 39). Furthermore, the bacterially expressed hOGA HAT-like domain does not bind acetyl-CoA *in vitro* (38).

Although strides have been made toward identifying the processes regulated by *O*-GlcNAcylation, uncovering the consequences of *O*-GlcNAc on individual proteins at an organismal level remains a challenge. Gene knock-out is a useful strategy to addressing the challenge by generating animals lacking OGT/OGA activity. However, the fact that *Ogt* null mice and *Drosophila ogt* mutants die at different stages of development and *Oga* null mice as neonates (30, 40, 41) limits their use for functional studies. Whereas levels of OGT and OGA have been manipulated in zebrafish embryos and *Xenopus laevis* oocytes to study the roles of *O*-GlcNAc in development (42, 43), knockouts of the enzymes have not been reported in these organisms. *C. elegans* is the only known example of an organism that remains viable and fertile after loss of OGT and OGA activity (29, 35). *ogt* and *oga* null mutants of *C. elegans* have therefore been used to study the effects of *O*-GlcNAc cycling on lifespan and aging (44–46). Accessible reductionist models with smaller *O*-GlcNAc proteomes are thus invaluable toward accelerating research into understanding the conserved roles and mechanisms of protein *O*-GlcNAcylation. The aim of this study was to find another such model.

Here, we report that the basal metazoan *Trichoplax adhaerens* is the simplest organism to possesses both OGT and OGA and *O*-GlcNAcylated proteins. OGT appears to be expressed throughout the body of *Trichoplax* under basal conditions. *Trichoplax* OGT can rescue pupal lethality of the *Drosophila sxc* (*ogt*) mutant in addition to compensating for the maternal requirement of OGT. *Trichoplax* OGA can de-*O*-GlcNAcylate human and *Drosophila* cell lysates. Together, these data imply that the acquisition of OGA by metazoa at the time of diverging from their unicellular ancestors facilitated the cycling of *O*-GlcNAc on proteins. This acquisition may have expanded the repertoire of complex signaling mechanisms required for metazoan-specific features absent in other intracellular OGT-possessing organisms lacking OGA.

## EXPERIMENTAL PROCEDURES

### 

#### 

##### Sequences and Alignments

Orthologues of OGA and OGT in *Trichoplax* were identified by using BLAST in the Uniprot database and the *Trichoplax* genome database. Query sequences were from the following: *Homo sapiens, Mus musculus, Danio rerio, Drosophila melanogaster*, and *C. elegans*. Sequences were aligned using CLUSTALW, and edited and annotated with ALINE. XtalPred and sequence alignments with *Og*OGA were used to predict regions of structural disorder in hOGA, *Dm*OGA and the *Ta*OGAs. Surface views of hOGT and *Og*OGA were generated and colored by similarity to their *Trichoplax* counterparts using PyMOL.

##### T. adhaerens Culture and Harvest

Starter cultures of *T. adhaerens* and the cryptomonad marine red alga *Rhodomonas salina*, which serves as a food source for *Trichoplax*, were obtained from Prof. Leo Buss (Yale University). *Trichoplax* were seeded and grown on a mat of monoculture of *Rhodomonas* in 150-mm glass Petri dishes at 22 °C in artificial seawater (Reef Crystals, Aquarium Systems) of 36 parts per thousand (4.5 brix %) salinity supplemented with 0.1% (v/v) Micro Algae Grow (Florida Aqua Farms). To harvest *Trichoplax*, culture medium in Petri dishes was gently pipetted up and down several times to lift adherent animals off the glass surface. The contents of the dish were then centrifuged at 1000 × *g* at 4 °C for 10 min. The algae were removed by washing with unsupplemented artificial seawater by repeated centrifugation at low speed.

##### Rapid Amplification of cDNA Ends (RACE)

*Trichoplax* total RNA was extracted using TRI reagent (Sigma). cDNA was synthesized using Precision qScript^TM^ Reverse Transcription kit (Primer Design) and an oligo(dT) primer or the FirstChoice® RLM-RACE Kit (Ambion). Full-length coding sequences for *Trichoplax* OGA and OGT were determined using the FirstChoice® RLM-RACE Kit (Ambion) according to the manufacturer's instructions. PCR products were gel purified and sequenced. Full-length sequences were then amplified from cDNA and cloned into pCR®-Blunt II-TOPO® (Invitrogen) for sequence verification. Two to four colonies were sequenced using both the M13-F and M13-R primers.

##### Cloning and Site-directed Mutagenesis

*Ta*OGA53 and *Ta*OGA54 were cloned into pGEX6P1 and pOPTH, respectively, using a previously described restriction-free method (47) from TOPO clones after RACE experiments identified start and end sites. N-terminally truncated *Ta*OGT was initially cloned through PCR amplification followed by BamHI-SalI digestion and ligation into pGEX6P1. Following identification of the start of *Ta*OGT through RACE experiments, a missing segment was added to the existing construct by the restriction free cloning method. Site-directed mutations were introduced using the Stratagene QuikChange Site-directed mutagenesis kit except KOD Polymerase (Novagen) was used instead of *Pfu*, and DpnI was purchased from Fermentas. The presence of the intended mutations was confirmed by DNA sequencing.

##### Protein Expression and Purification

Plasmids containing *Ta*OGT and *Ta*OGA53 were transformed into *Escherichia coli* ArcticExpress competent cells (Stratagene), whereas *Ta*OGA54 and hCK2α were transformed into *E. coli* BL21(DE3) pLysS cells. Cells were grown overnight at 37 °C in Luria-Bertani medium containing 50 μg/ml of ampicillin (LB-Amp) and used at 10 ml/liter to inoculate 6 liters of fresh LB-Amp in the case of BL21(DE3) pLysS cells and 12 liters for ArcticExpress cells. BL21(DE3) pLysS cells were grown to an *A*_600_ of 0.6–0.8, transferred to 18 °C, and induced with 250 μm isopropyl 1-thio-β-d-galactopyranoside and harvested after 16 h. ArcticExpress cells were grown to an *A*_600_ = 1.0, transferred to 12 °C, and induced with 250 μm isopropyl 1-thio-β-d-galactopyranoside and harvested after 72 h by centrifugation for 30 min at 3500 rpm (4 °C). Cell pellets were resuspended in 10–20 ml/liter of 50 mm Tris, 250 mm NaCl, and 0.5 mm Tris(2-carboxyethyl)phosphine (lysis buffer) at pH 9.0 for *Ta*OGT and hCK2α and pH 7.5 for *Ta*OGA53 and *Ta*OGA54. Lysis buffers for *Ta*OGT and *Ta*OGA53 also contained 5% glycerol and 0.05% Nonidet P-40. All lysis buffers were supplemented with protease inhibitors (1 mm benzamidine, 0.2 mm PMSF, and 5 μm leupeptin), DNase and lysozyme prior to lysis. Cells were lysed using a continuous flow cell disrupter (Avestin, 3 passes at 20 kpsi) and the lysate was cleared by centrifugation (30 min, 15,000 rpm, 4 °C). Supernatants were collected and loaded onto 2 ml of glutathione-Sepharose (GE Healthcare Life Sciences) pre-equilibrated with lysis buffer. *Ta*OGA54 was loaded on to 2 ml of IMAC Sepharose (GE Healthcare) charged with NiSO_4_ and pre-equilibrated with lysis buffer. Loaded resins were each washed with 500 ml of lysis buffer or lysis buffer containing 30 mm imidazole in the case of IMAC resin. The ArcticExpress chaperones were removed from captured GST-tagged proteins by washing the resin with 1× TBS (25 mm Tris, pH 7.5, 150 mm NaCl) containing 10 mm ATP and 11 mm MgCl_2_ (4× washes at 37 °C). GST- tagged proteins were eluted from resin by cleavage of the GST tag using GST-tagged PreScission^TM^ protease at 4 °C for 16 h. His_6_-tagged *Ta*OGA54 was eluted using lysis buffer containing 250 mm imidazole and dialyzed into 1× TBS containing 0.5 mm Tris(2-carboxyethyl)phosphine. *Ta*OGA54 and hCK2α were further purified by size exclusion chromatography using a Superdex 200, 26/60 column. All proteins were concentrated using spin concentrators and purity was assessed by SDS-PAGE followed by Coomassie R-250 staining. Point mutants of *Ta*OGT, *Ta*OGA53, and *Ta*OGA54 were purified the same way as their wild type counterparts.

##### Steady-state Kinetics

*K_m_* for UDP-GlcNAc of wild type and mutant *Ta*OGT was determined as described previously (48). Briefly, 100 μl reactions contained 100 nm *Ta*OGT in 50 mm Tris, pH 7.5, 0.1 mg/ml of BSA, 10 μm sodium dithionite, and 100 μm peptide (KKENSPAVTPVSTA) and varying amounts of UDP-GlcNAc. Reactions were carried out for 75 min at room temperature and stopped using 200 μl of 37.5 μm fluorophore (48–50) prepared in 50 mm HEPES, pH 7.5, 10 mm NaCl, and 50% (v/v) methanol. Fluorescence was measured using Gemini EM plate reader (Molecular Devices) with excitation and emission wavelengths of 485 and 530 nm, respectively. The IC_50_ for Goblin 1 was determined using 13 μm UDP-GlcNAc, 100 μm peptide, and varying concentrations of the inhibitor. Steady-state kinetics of wild type and mutant *Ta*OGA54 and *Ta*OGA53 were determined as described (51) using 4-methylumbelliferyl-*N*-acetyl-β-d-glucosaminide (4MU-NAG, Sigma). Reaction mixtures (100 μl) contained 2–100 nm enzyme in 1× TBS, 0.1 mg/ml of BSA, and varying amounts of substrate in 1–2% dimethyl sulfoxide. Reactions were performed for 30–120 min at room temperature and stopped by the addition of 200 μl of glycine-NaOH, pH 10.3. Fluorescence of released 4-methylumbelliferone was measured using a Synergy 2 plate reader (Bio-Tek), with excitation and emission wavelengths of 360 and 460 nm, respectively. IC_50_ values were measured at *K_m_* with varying concentrations of inhibitors. All experiments were performed in triplicate and measurements were corrected for background emission from reactions containing no peptide (for OGT assays) or no enzyme (for OGA assays). For all assays performed, substrate turnover was under 10%. Non-linear regression curves were fitted with Prism (GraphPad).

##### In Vitro O-GlcNAcylation of hCK2α

Reactions contained 0.25 μg of hCK2α, 3.7 mm UDP-GlcNAc, and 2.5 μm of either hOGT/GST-hOGT (purified as described previously (52)) or *Ta*OGT in a total volume of 10 μl of 10 mm Tris, pH 7.5, and 1 mm DTT and incubated at room temperature for 1.5 h. For subsequent *Ta*OGA treatments, GST-hOGT was pulled out of the reactions using glutathione-Sepharose and residual hOGT activity was blocked using 5 mm UDP. Reactions were stopped by the addition of Laemmli buffer and proteins were separated by SDS-PAGE and analyzed by Western blotting as described below.

##### Drosophila Genetics and Adult Fly Lysates

The following stocks were used: *w*^1118^, *sxc*^1^/*CyO, sxc*^6^/*CyO*, and tub::GAL4/TM6. Transgenic flies were generated by Rainbow Transgenic Flies Inc., CA, with the attP insertion site at 86F8. 5 anesthetized male adult flies were frozen on dry ice and homogenized in 50 μl of lysis buffer (50 mm Tris-HCl, pH 8.0, 150 mm NaCl, 1% Triton X-100, 1 μm GlcNAcstatin C, 5 mm sodium fluoride, 2 mm sodium orthovanadate, 1 mm benzamidine, 0.2 mm PMSF, 5 μm leupeptin, and 1 mm DTT), following which an equal volume of 3× SDS Laemmli buffer was added. Lysates were then boiled for 5 min at 95 °C, centrifuged at 16,000 × *g* for 10 min, and supernatants were collected. 30 μg of crude lysates were used for Western blots.

##### Cell Culture, Lysis, and Protein Extraction

HEK293 cells were maintained in DMEM (Invitrogen) supplemented with 10% FBS (Gibco) and antibiotics (Gibco) at 37 °C in a humidified atmosphere. *Drosophila* S2 cells were cultured in Schneider's medium supplemented l-glutamine, 10% FBS (Gibco), and antibiotics (Gibco) at 25 °C. HEK293, S2 cells, and *Trichoplax* were lysed in 10 mm Tris, pH 7.5, 150 mm NaCl, 1% Nonidet P-40 supplemented with protease inhibitors (1 mm benzamidine, 0.2 mm PMSF, and 5 μm leupeptin). Transfected S2 cells were lysed with 50 mm Tris-HCl, pH 8.0, 150 mm NaCl, 1% Triton, 1 μm GlcNAcstatin C, 1 mm sodium orthovanadate, 5 mm sodium fluoride, and protease inhibitors. Lysates were cleared by centrifugation. Bradford assay or a Pierce 660-nm protein assay was used to quantify cell lysates.

##### Transfections, RNAi, and Enzymatic Treatments of Lysates

S2 cell transfections were carried out by mixing FuGENE HD (Roche), DNA (2 μg) at a 3:2 ratio (μl:μg) in 100 μl of sterile water. The constructs used for transfections were pMT-GAL4, pUAS-*Dm*OGT^WT^-HA, pUAS-*Ta*OGT^WT^-HA, and pUAS-*Ta*OGT^K815M^-HA. The metallothionein promoter was induced with 1 mm CuSO_4_ 24 h after transfection. RNAi was performed 48 h before DNA transfections by transfecting 4 μg of double-stranded RNA directed against the 3′ UTR of *Dm*OGT transcript. Double-stranded RNA was synthesized using a TranscriptAid T7 High Yield Transcription Kit (Thermo) according to the manufacturer's instructions from PCR products containing T7 (indicated in lowercase in the primer sequences) sites introduced by the following primers: forward, taatacgactcactatagggAAAACGTTTATAATGTCAAT and reverse, taatacgactcactatagggTTCTTATTATATATCGTATA.

20 μg of lysates were subjected to all enzymatic treatments. PNGase F (New England Biolabs) treatment was performed as described by the manufacturer. *Cp*NagJ (purified as described in Ref. 51, but with the GST tag left uncleaved at the N terminus) treatment was performed with 2–4 μg of the enzyme at 37 °C for 1 h. *Ta*OGA54 and *Ta*OGA53 treatments were performed on lysates and *in vitro O*-GlcNAcylated hCK2α with 5, 10, or 15 μg of the enzymes for 4 h at room temperature. Labeling of lysates with GalT1 (Y289L) was performed according to the manufacturer's instructions (Invitrogen).

##### Western Blotting

Proteins were resolved in SDS-PAGE gels and blotted onto nitrocellulose or PVDF membranes. Polyclonal antibodies were generated by immunizing rabbits (Dundee Cell Products) with a pair of peptides from each protein (*Ta*OGT, EYADHYSEKLAFLPNS and TRLRKLQDKIWQLRHKC; *Ta*OGA53, HKYGRHSIHLINMARC and TEATKHSSDATDTVDSC; and *Ta*OGA54, LYLSHLEARFDSSVPEK and CRFILEQLKAKGSYGAS) and affinity purified using a 1:1 mixture of the peptide antigens coupled to NHS-activated agarose (Thermo). The following antibodies were used: anti-*O*-GlcNAc CTD110.6 (1:500, Covance), anti-*O*-GlcNAc RL-2 (1:3,000, Abcam), anti-actin (1:5,000, Sigma), anti-OGT DM-17 (1:5,000, Sigma), anti-OGT H-300 (1:1,000, Santa Cruz), anti-HA (12CA5, 1:2,500) and anti-CK2α (1:5,000, Cell Signaling), anti-*Ta*OGT (1:5,000 of ∼8 μg/ml), anti-*T*aOGA53 (1:5,000 of ∼60 μg/ml), and anti-*Ta*OGA54 (1:5,000 of ∼8 μg/ml). HRP-conjugated anti-mouse IgM and IgG, anti-rabbit IgG, and ExtrAvidin®-peroxidase were purchased from Sigma. Biotin-conjugated concanavalin A was also purchased from Sigma and used as per the manufacturer's instructions. Anti-mouse Alexa Fluor® 680 and anti-rabbit Alexa Fluor® 790 were purchased from Jackson ImmunoResearch. Secondary antibodies were used at dilutions of 1:10,000 or 1:20,000. Blots were developed using ECL or imaged using the Li-Cor Odyssey infrared imaging system (Li-Cor). Blots with *Trichoplax* lysates were stained with Coomassie R-250 or Ponceau S for total protein loading.

##### In Situ Hybridization

*Trichoplax* were fixed as described (53) with slight modifications. Briefly, the animals were fixed using Lavdovsky's fixative (20% formaldehyde, 4% glacial acetic acid, 32% of 10× PBS, and 44% ethanol (v/v) in deionized water), washed, and permeabilized using PBST (1× PBS containing 0.1% Tween 20). Dioxigenin-labeled probes were synthesized using TranscriptAid T7 High Yield Transcription Kit (Thermo) according to the manufacturer's instructions from PCR products containing T7 (indicated in lowercase in the primer sequences) sites introduced to the sense or antisense strands by primers: sense forward, taatacgactcactatagggCGCCATGGAAATCTTTGTTT, sense reverse, TCTGCGTATTCCATTGGTGA; antisense forward, CGCCATGGAAATCTTTGTTT, antisense reverse, taatacgactcactatagggTCTGCGTATTCCATTGGTGA.

Prehybridization and hybridization were carried out in 2.5× SSC buffer containing 50% formamide, 5% dextran sulfate, and 0.1 mg/ml of yeast tRNA. Approximately 0.5 μg of probes were used per 1 ml of hybridization buffer and hybridizations were carried out overnight for each probe based on length of the probe and GC content at the temperature determined by the following formula,

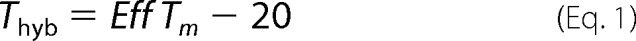
 where Eff *T_m_* = 81.5 + 16.6(log *M* [Na^+^]) + 0.41(%*G* + *C*) − 0.72(% formamide).

Samples were then washed at low stringency using 100 mm maleic acid-buffered saline at pH 7.5 containing 0.1% Tween 20 (MABT). High stringency washes were performed with hybridization buffer at 98% stringency at temperatures determined using the following formula.

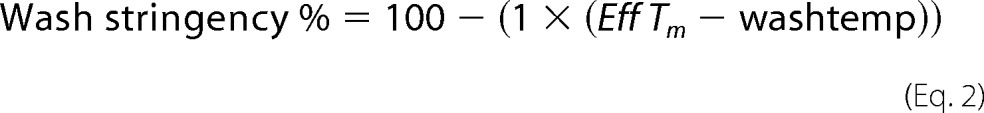
 Samples were again washed in MABT, blocked with blocking buffer (3% BSA in MABT), and incubated in a 1:2000 dilution (in blocking buffer) of alkaline phosphatase-conjugated anti-digoxigenin Fab fragments (Roche Diagnostics) for 5 h at room temperature. Following washes with MABT and deionized water, the color reaction was carried out using the alkaline phosphatase substrate BM purple (Roche Diagnostics). Samples were then mounted on slides with Vectashield medium-set mountant (Vector Biolabs) and imaged using a Leica DM2000 microscope (Leica Microsystems).

## RESULTS

### 

#### 

##### Trichoplax Expresses Orthologues of Metazoan OGT and OGA

We aimed to identify basal organisms possessing both OGT and OGA genes in an attempt to identify a reductionist model to probe *O*-GlcNAc signaling and shed light on the evolution of reversible intracellular protein *O*-GlcNAcylation. Reports have suggested the presence of *O*-GlcNAcylated proteins in filamentous fungi (54), protists (55, 56), and bacteria (57, 58). Plants and primitive eukaryotes possessing apparent OGT orthologues and *O*-GlcNAcylated proteins appear to lack OGA (55, 56, 59), suggesting that *O*-GlcNAcylation is either irreversible in these organisms, or may be reversed by unidentified enzymes bearing no similarity to metazoan OGA. Conversely, in the bacteria in which *O*-GlcNAc has been found, bioinformatics searches did not identify OGT-like proteins. We parsed the CAZy database, which lists a number of other organisms ranging from archaea to man that possess enzymes of the glycosyltransferase family 41 (GT41) and the glycoside hydrolase family 84 (GH84) to which OGT and OGA, respectively, belong. Upon close examination, it is clear that of all these organisms, only metazoa possess clear orthologues of both OGT and OGA (bearing over 40% sequence identity to hOGT and hOGA) in their genomes. We then searched the genomes of basal metazoa and identified an OGT gene fragment and two candidate OGA gene fragments in the recently sequenced genome of *T. adhaerens* (60), the sole member of phylum placozoa. *Trichoplax* is a free-living marine organism considered to be one of the most basal extant multicellular organisms existing at the boundary between unicellular eukaryotes and metazoa (60–62). It contains only six cell types organized in three cellular layers (61, 63). The presence of putative stem cells at the periphery of the body of *Trichoplax* has been hypothesized (53), but remains unconfirmed (63).

**FIGURE 1.**
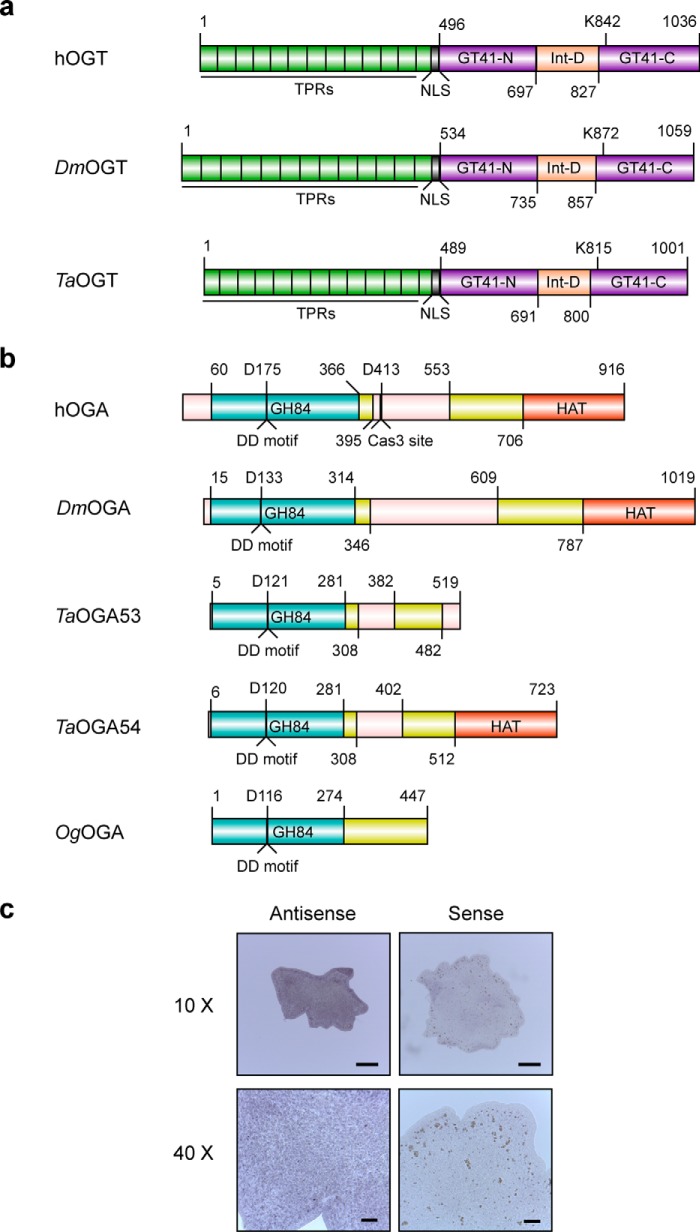
***Trichoplax* possesses OGT and OGA orthologues.** *a,* schematic showing the domains of *Ta*OGT compared with hOGT and *Dm*OGT. The TPR region of the proteins is shown in *green*, the N- and C-terminal catalytic lobes (GT41-N and GT41-C) in *purple*, and the intervening domain (*Int-D*) in *peach*. The conserved lysine residue required for catalytic activity of the OGTs is shown, as is the conserved nuclear localization signal (*NLS*) in the proteins. *b,* schematic showing the domain architecture of the *Ta*OGAs compared with hOGA, *Dm*OGA, and *Og*OGA (a structurally characterized bacterial OGA with high sequence conservation with hOGA (66)). The catalytic domain with glycoside hydrolase activity (GH84) is shown in *blue*, the middle domain in *yellow*, and the HAT-like domain (HAT) in *orange*. The Asp-Asp motif (*DD motif*) required for activity and the catalytic residue within it are depicted. The caspase 3 cleavage site of hOGA is also shown. Regions of predicted structural disorder within the proteins are shown in *pale salmon. c,* localization of *Ta*OGT transcripts analyzed by whole mount *in situ* hybridization using dioxigenin-labeled probes. Hybridized probes detected using alkaline phosphatase (*AP*)-conjugated anti-dioxigenin antibody and the AP substrate BM purple. *Purple* staining throughout the organism shows ubiquitous presence of *Ta*OGT transcripts. *Scale bars*: *top panels*, 100 μm; *bottom panels*, 20 μm.

Upon identifying fragments of *ogt* and *oga* genes in *Trichoplax* using bioinformatics, we performed 5′ and 3′ RACE (rapid amplification of cDNA ends) to obtain full-length sequences of these genes. It emerged that *Trichoplax* OGT (*Ta*OGT), apart from having the catalytic domain, contains 13.5 N-terminal TPR repeats and a putative bipartite nuclear localization signal like hOGT (64) (Fig. 1*a*, supplemental Fig. S1*a*). It shares 66 and 64% overall amino acid sequence identity with hOGT and *D. melanogaster* OGT (*Dm*OGT), respectively (Fig. 1*a*, supplemental Fig. S1*a*). Its active site is conserved with that of hOGT (supplemental Fig. S1*b*), and contains the key lysine residue (Lys-815 in *Ta*OGT and Lys-842 in hOGT- Fig. 1*a*, supplemental Fig. S1*a*), shown to be critical for the activity of hOGT (52). The most variable region is the intervening domain within the catalytic lobes of the enzyme, whereas the TPRs are the most conserved (supplemental Fig. S1*a*). Unlike other metazoa, which possess a single *oga* gene, the genome of *Trichoplax* encodes for two putative OGAs, *Ta*OGA53 and *Ta*OGA54 (after their Uniprot IDs B3SB53 and B3SB54). *Ta*OGA53 resembles the shorter hOGA isoform lacking the HAT-like domain, whereas *Ta*OGA54 is similar to the full-length hOGA (Fig. 1*b*, supplemental Fig. S2*a*). The glycoside hydrolase domains of *Ta*OGA53 and *Ta*OGA54 share 60% sequence identity with each other and are over 50% identical in amino acid composition to the glycoside hydrolase domain of hOGA and *D. melanogaster* OGA (*Dm*OGA). The Asp-Asp motif shown to be important for hOGA activity (65) is conserved in the *Ta*OGAs (Fig. 1*b*, supplemental Fig. S2*a*). The *Ta*OGAs are also both about 40% identical to the structurally characterized bacterial OGA from *Oceanicola granulosus* (*Og*OGA) (66), with which they share a conserved active site (Fig. 1*b*, supplemental Fig. S2, *b* and *c*). Neither *Ta*OGA53 nor *Ta*OGA54 appears to have a caspase 3-cleavage site, a feature present in hOGA (Fig. 1*a*, supplemental Fig. S2*a*) (37).

For immunodetection of *Ta*OGT and the *Ta*OGAs, antibodies were raised against two unique peptides within each protein. However, antigen-purified antibodies could only detect their respective recombinant proteins and not endogenous *Ta*OGT or *Ta*OGA53 and *Ta*OGA54 in lysates by Western blotting or immunoprecipitation. This was probably either due to low expression levels of these proteins or weak affinity of the antibodies toward them. Nevertheless, analysis of published high-resolution proteomics reveals the presence of *Ta*OGT and *Ta*OGA54 in the *Trichoplax* proteome when the organism is cultured under standard culture conditions (67).

Reports have suggested the existence of a ring of putative stem cells at the periphery of the body of *Trichoplax* (53), where specific orthologues of the developmental genes *Hox*, *T-box*, and *Pax* have been shown to be expressed (53, 67–70). Given that *Ogt* is part of the polycomb group of developmental genes in *Drosophila* (30) and has been shown to be essential for stem cell viability in mice (40), we wanted to investigate localization of OGT in *Trichoplax* to explore its potential function in this basal metazoan. In the absence of a robust antibody for immunofluorescence staining, we performed *in situ* hybridization to localize OGT transcripts in *Trichoplax* whole mounts. Dioxigenin-labeled antisense probes were used and sense controls were performed in parallel. To detect hybridized probes, we used alkaline phosphatase-conjugated anti-dioxigenin antibodies and a substrate that turns purple upon reacting with alkaline phosphatase. In contrast to the transcripts of the aforementioned developmental genes, OGT transcripts were not restricted to the periphery of the organism. Instead, we observed that OGT transcripts were distributed evenly in the organism in samples probed with antisense RNA (*n* = 12) with negligible staining in those probed with the sense control (*n* = 12) (Fig. 1*c*). This pattern is similar to the expression of the ubiquitous actin (53) and suggests that OGT, which is expressed in several tissues in higher organisms (71), may also be expressed and have functions in the different cell types of a basal organism like *Trichoplax*.

##### TaOGT Is a Functional O-GlcNAc Transferase

**FIGURE 2.**
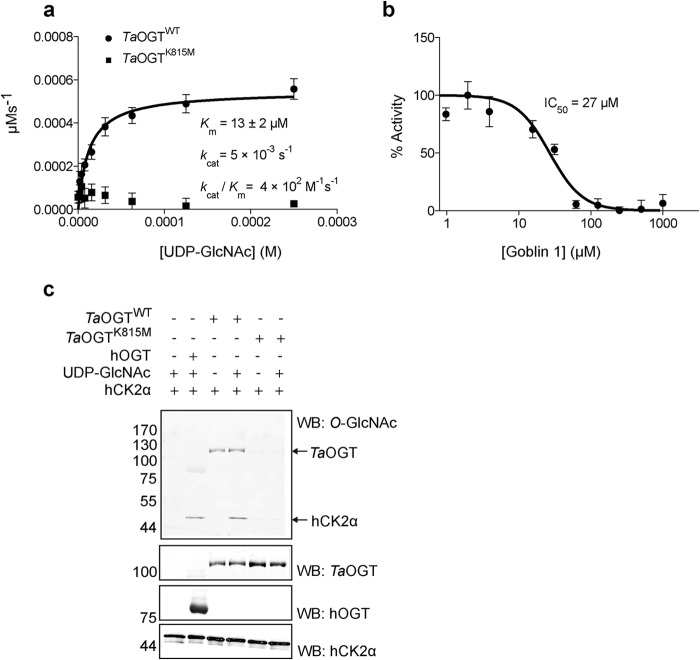
***Ta*OGT is a functional *O-*GlcNAc transferase.** *a,* Michaelis-Menten kinetics of *Ta*OGT measured using the peptide substrate KKENSPAVTPVSTA and varying amounts of UDP-GlcNAc. Reactions were carried out for 75 min at room temperature and read after the addition of a compound that fluoresces upon binding to the reaction product UDP (48–50). Data points were fitted to the Michaelis-Menten equation using Prism (GraphPad). Experiments were performed in triplicate and *error bars* represent mean ± S.E. *b,* IC_50_ of the bisubstrate inhibitor Goblin 1 for *Ta*OGT. IC_50_ was measured using UDP-GlcNAc at a concentration equal to the *K_m_* and varying amounts of the inhibitor. Highest activity in the absence of inhibitors is arbitrarily set as 100%. Data points were fitted to a three-parameter equation for dose-dependent inhibition using Prism (GraphPad). Experiments were performed in triplicate and *error bars* represent mean ± S.E. *c, in vitro O-*GlcNAcylation of hCK2α by *Ta*OGT and autoglycosylation of *Ta*OGT detected by Western blotting using the anti-*O*-GlcNAc antibody RL-2. hCK2α was incubated with *Ta*OGT and a molar excess of UDP-GlcNAc at room temperature for 1.5 h. Negative controls include hCK2α treated with the catalytically inactive *Ta*OGT^K815M^ or with *Ta*OGT^WT^ in the absence of the donor substrate UDP-GlcNAc. hCK2α treated with hOGT(312–1031) was used as a positive control. *WB*, Western blot.

We cloned, recombinantly expressed, and purified the putative *Ta*OGT to investigate its activity and elucidate its biochemical properties. The negative control for this experiment was the Lys-815 (*Ta*OGT^K815M)^ mutated to Met because the equivalent Lys-842 residue in hOGT is indispensable for catalysis (52). Steady-state kinetics were performed employing a recently published fluorescence assay (48). The *K_m_* for UDP-GlcNAc was measured in the presence of excess peptide substrate (KKENSPAVTPVSTA, previously used as a substrate to measure the activity of hOGT (48)) and was found to be 13 ± 2 μm, within the range reported for hOGT (24, 72–74) (Fig. 2*a*). *Ta*OGT activity is inhibited by the OGT bisubstrate inhibitor Goblin 1 (48) with an IC_50_ (27 μm) comparable with that reported for hOGT (Fig. 2*b*). Furthermore, *Ta*OGT^WT^, but not the inactive mutant *Ta*OGT^K815M^, could *O*-GlcNAc modify human CK2α (hCK2α), a well characterized hOGT substrate (75–77) *in vitro*, thus validating it as a true OGT orthologue (Fig. 2*c*). This experiment also revealed that *Ta*OGT^WT^, like its full-length human counterpart (75), undergoes autoglycosylation as evidenced by the reactivity of the anti-*O*-GlcNAc antibody RL-2 toward *Ta*OGT^WT^ but not *Ta*OGT^K815M^ (Fig. 2*c*).

##### TaOGT Rescues Drosophila supersex combs (sxc) Lethality

**FIGURE 3.**
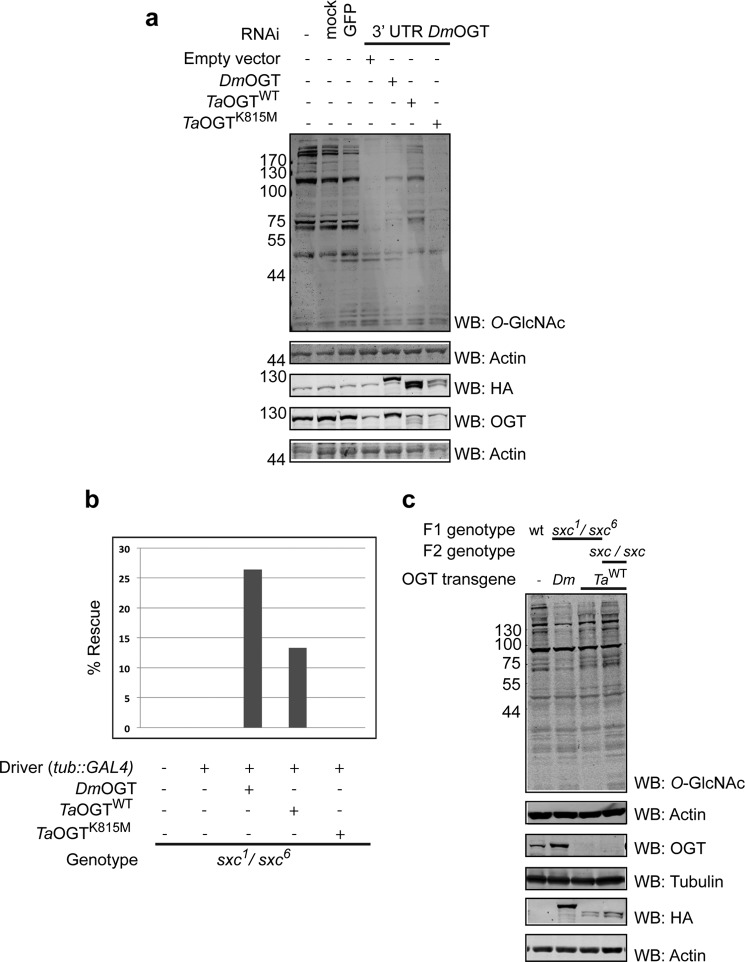
***Ta*OGT can rescue the lethality of *Drosophila supersex combs* (*sxc*) mutants.** *a, Ta*OGT^WT^ restores *O-*GlcNAc levels in S2 cells lacking endogenous OGT. RNAi was used to knockdown endogenous OGT in S2 cells. GFP RNAi was used as a control. Cells were then transfected with plasmids carrying HA-tagged *Dm*OGT, *Ta*OGT^WT^, or catalytically inactive *Ta*OGT^K815M^. Cells were lysed and total lysates were probed by Western blotting using the specified antibodies. *b,* quantification of rescue to adulthood on driving *Dm*OGT, *Ta*OGT^WT^, or *Ta*OGT^K815M^ transgenes in *sxc*^1^/*sxc*^6^ mutants. The number of *sxc* transheterozygotes recovered on rescue with *Ta*OGT^WT^ is comparable with that of the *Dm*OGT. The level of rescue with *Dm*OGT is twice as that of *Ta*OGT^WT^ because the transgenic line used in the case of *Dm*OGT was homozygous. *c, O-*GlcNAc levels in flies expressing *Ta*OGT^WT^ are comparable with those expressing *Dm*OGT. Total lysates from *w*^1118^ (wt), rescued F1 *sxc*^1^/*sxc*^6^ transheterozygotes, or F2 *sxc/sxc* flies expressing HA-tagged UAS::*DmOGT* or UAS::*Ta*OGT^WT^ under the control of tubulin::GAL4 were probed by Western blotting using the specified antibodies. *WB*, Western blot.

*Drosophila* OGT mutants, also known as *supersex combs (sxc*) mutants, die as pharate adults and this lethality can be rescued by ubiquitous expression of transgenic wild type *Dm*OGT in *sxc* transheterozygotes (31). We used this approach to investigate the functional equivalence of *Ta*OGT and *Dm*OGT. Initial experiments were performed in S2 cells where endogenous OGT was knocked down using RNAi directed towards the 3′ UTR of *Dm*OGT and cells were transfected with plasmids carrying either *Ta*OGT^WT^ or the catalytically inactive *Ta*OGT^K815M^. *O*-GlcNAc levels in cells transfected with *Ta*OGT^WT^, but not *Ta*OGT^K815M^, were restored to levels comparable with cells transfected with *Dm*OGT (Fig. 3*a*). Strikingly, in the context of the whole organism, the number of *sxc* transheterozygotes recovered on rescue with *Ta*OGT (13% of total progeny) was comparable with that of the *Dm*OGT (26% of total progeny). The level of rescue with *Dm*OGT is twice as that of *Ta*OGT^WT^ because the transgenic line used in the case of *Dm*OGT was homozygous (Fig. 3*b*, Table 1). In the control crosses lacking either the driver or the transgene, no adult *sxc*^1^/*sxc*^6^ transheterozygotes were recovered. *sxc* is a maternal effect gene and the rescue by *Ta*OGT to produce F1 progeny is the rescue of the zygotic requirement of OGT. To assess whether the maternal OGT function could also be rescued by *Ta*OGT, the rescued F1 males were crossed with rescued F1 females. The only completely functional OGT in this cross is the *Ta*OGT^WT^ driven by tubulin::GAL4. Fertile F2 progeny were recovered from this cross establishing that *Ta*OGT could also substitute for the maternal requirement of *sxc* in early *Drosophila* development. The catalytically inactive *Ta*OGT^K815M^, on the other hand, does not rescue the *sxc* lethality phenotype. The level of rescue of the catalytic activity of *Ta*OGT was investigated by performing Western blots against total *O*-GlcNAc using adult fly lysates. The level of total *O*-GlcNAc both in F1 and F2 *Ta*OGT^WT^ rescued *sxc* adults is comparable with that of *Dm*OGT rescued *sxc* mutants (Fig. 3*c*), implying that *Ta*OGT is a fully functional OGT orthologue.

**TABLE 1:** **Rescue of *sxc* lethality by *Ta*OGT** Crosses were setup up with flies of the indicated genotypes and transferred into fresh vials every 3–4 days. Adults emerging from the crosses were scored for the presence of second and third chromosome balancers/marker, CyO and MKRS or TM6. Flies that did not possess any of the balancers/markers (+; +) were the rescued *sxc^1^/sxc^6^* transheterozygotes. Control crosses with flies lacking either the driver (tubulin::GAL4) or any of the OGT transgenes do not yield any non-CyO adults.

Parental cross	Total adults	CyO; TM6	CyO; MKRS	CyO; MKRS/TM6	CyO;+	+; +
*sxc^6^/CyO; tub::GAL4/TM6*♀ × *sxc^1^/CyO;MKRS/TM6*♂	119	26	54	39	NA*^a^*	0
*sxc^6^/CyO;MKRS/TM6*♀ × *sxc^1^/CyO;UAS::TaOGT^WT^/TM6* ♂	195	54	97	44	NA	0
*sxc^6^/CyO;tub::GAL4/TM6*♀ × *sxc^1^/CyO;UAS::DmOGT^WT^* ♂	174	76	NA	NA	52	46
*sxc^6^/CyO;tub::GAL4/TM6*♀ × *sxc^1^/CyO;UAS::TaOGT^WT^/TM6* ♂	203	120	NA	NA	50	27
*sxc^6^/CyO;tub::GAL4/TM6*♀ × *sxc^1^/CyO;UAS::TaOGT^K815M^/TM6* ♂	131	94	NA	NA	37	0

*^a^* NA, not applicable.

##### TaOGA53 and TaOGA54 Are Functional O-GlcNAcases

**FIGURE 4.**
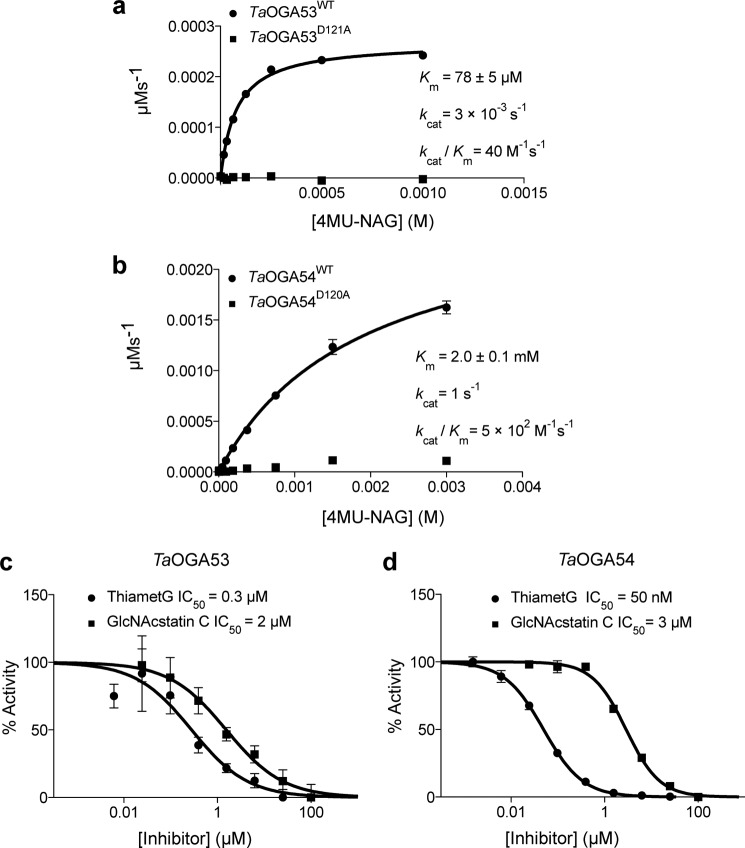
***Ta*OGA53 and *Ta*OGA54 are functional *O-*GlcNAcases.** *a,* Michaelis-Menten kinetics of *Ta*OGA53 using varying amounts of 4MU-NAG. Reactions were carried out for 120 min at room temperature. Data points were fitted to the Michaelis-Menten equation using Prism (GraphPad). Experiments were performed in triplicate and *error bars* represent mean ± S.E. *b,* Michaelis-Menten kinetics of *Ta*OGA54 measured using varying amounts of the fluorescent substrate 4MU-NAG. Reactions were carried out for 30 min at room temperature. Data points were fitted to the Michaelis-Menten equation using Prism (GraphPad). Experiments were performed in triplicate and *error bars* represent mean ± S.E. *c*, IC_50_ of Thiamet G and GlcNAcstatin C for *Ta*OGA53. IC_50_ values were measured using 4MU-NAG at a concentration equal to the *K_m_* and varying amounts of inhibitors. Highest activity in the absence of inhibitors is arbitrarily set as 100%. Data points were fitted to a three-parameter equation for dose-dependent inhibition using Prism (GraphPad). Experiments were performed in triplicate and *error bars* represent mean ± S.E. *d,* IC_50_ of Thiamet G and GlcNAcstatin C for *Ta*OGA54. IC_50_ values were measured using 4MU-NAG at a concentration equal to the *K_m_* and varying amounts of inhibitors. Highest activity in the absence of inhibitors is arbitrarily set as 100%. Data points were fitted to a three-parameter equation for dose-dependent inhibition using Prism (GraphPad). Experiments were performed in triplicate and *error bars* represent mean ± S.E.

To test whether the putative *Ta*OGA53 and *Ta*OGA54 are active enzymes, we recombinantly expressed and purified them from *E. coli*. The negative controls were the Ala mutants of the predicted catalytic residues in the Asp-Asp motif (65) of the enzymes (Asp^120^, *Ta*OGA53^D120A^ and Asp^121^, *Ta*OGA54^D121A^). Steady-state kinetics experiments were performed using the fluorogenic pseudo-substrate 4MU-NAG. A Michaelis constant *K_m_* of 78 ± 5 μm and a turnover number *k*_cat_ of 3 × 10^−3^ s^−1^, were obtained for *Ta*OGA53, the shorter of the *Ta*OGAs (Fig. 4*a*), whereas *Ta*OGA54, the longer enzyme containing the HAT-like domain, was found to have a *K_m_* of 2.0 ± 0.1 mm and a turnover number *k*_cat_ of 1 s^−1^ (Fig. 4*b*). In contrast, the long hOGA isoform has been reported to have a lower *K_m_* than the shorter isoform (78, 79). The catalytic efficiency (*k*_cat_/*K_m_*) of *Ta*OGA54 (5 × 10^2^ m^−1^ s^−1^) is 12.5-fold higher than that of *Ta*OGA53 (40 m^−1^ s^−1^). The reduced catalytic activity indicated by the ∼300-fold decrease in *k*_cat_ of *Ta*OGA53 compared with *Ta*OGA54 is comparable with the reduced catalytic activity of the short *versus* full-length isoforms of hOGA reported previously (78, 79). The activities of both *Ta*OGA53 and *Ta*OGA54 are inhibited by the well characterized OGA inhibitors GlcNAcstatin C (80, 81) and Thiamet G (82) (Fig. 4, *c* and *d*).

**FIGURE 5.**
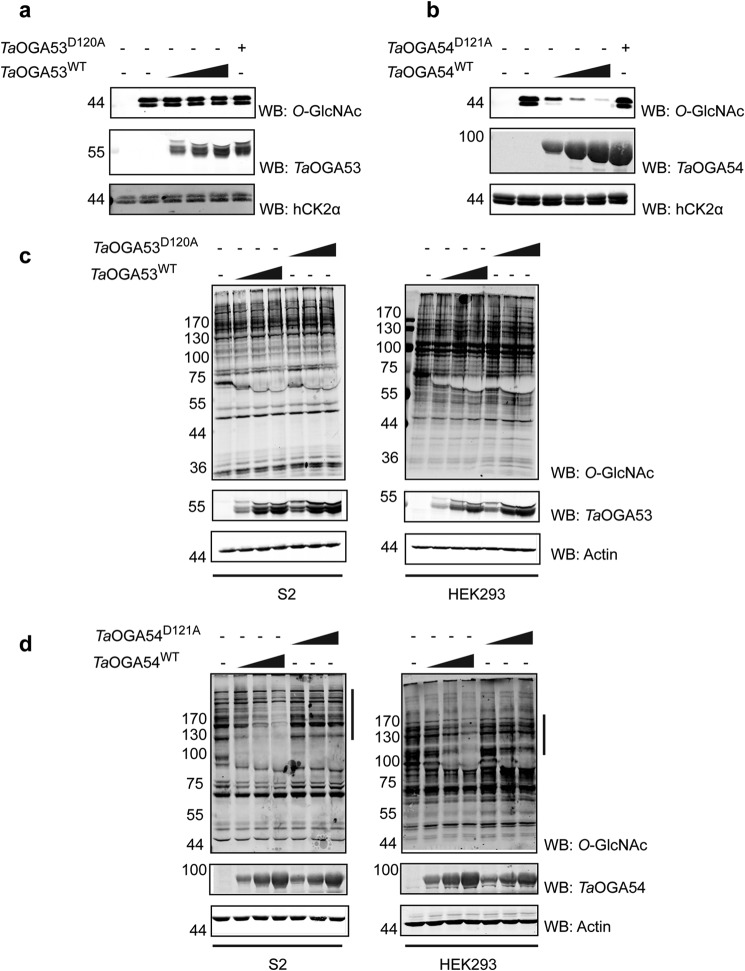
***In vitro* activity of *Ta*OGA53 and *Ta*OGA54.** *a,* activity of *Ta*OGA53 (5, 10, and 15 μg) on *in vitro O*-GlcNAcylated hCK2α detected by Western blotting using the anti-*O*-GlcNAc antibody RL-2. Reactions were carried out at room temperature for 4 h. A sample treated with 15 μg of the inactive mutant *Ta*OGA53^D120A^ was included as a negative control. Non-*O*-GlcNAcylated hCK2α (*first lane*) was also included as a negative control. *b,* activity of *Ta*OGA54 (5, 10, and 15 μg) on *in vitro O*-GlcNAcylated hCK2α detected by Western blotting using the anti-*O*-GlcNAc antibody RL-2. Reactions were carried out at room temperature for 4 h. A sample treated with 15 μg of the inactive mutant *Ta*OGA53^D120A^ was included as a negative control. Non-*O*-GlcNAcylated hCK2α (*first lane*) was also included as a negative control. *c,* activity of *Ta*OGA53 (5, 10, and 15 μg) on 20 μg of S2 and HEK293 cell lysates detected by Western blotting using the anti-*O*-GlcNAc antibody RL-2. Reactions were carried out at room temperature for 4 h. Lysates treated with the inactive mutant *Ta*OGA53^D120A^ were included as a negative control. *d,* activity of *Ta*OGA54 (5, 10, and 15 μg) on 20 μg of S2 and HEK293 cell lysates detected by Western blotting using the anti-*O*-GlcNAc antibody RL-2. Reactions were carried out at room temperature for 4 h. Lysates treated with the inactive mutant *Ta*OGA54^D121A^ were included as a negative control. Reduction in specific *O*-GlcNAc signal as a consequence of treatment with *Ta*OGA54^WT^ is indicated by *lines to the right* of the RL-2 blots.

To further confirm that *Ta*OGA53 and *Ta*OGA54 are active, *in vitro O*-GlcNAcylated hCK2α and HEK293/*Drosophila* S2 cell lysates were treated with increasing amounts of the enzymes and probed for *O*-GlcNAc using the RL-2 antibody. Samples were also treated with the catalytically inactive *Ta*OGA53^D120A^ and *Ta*OGA54^D121A^. Treatment of samples with *Ta*OGA53^WT^ did not lead to a noticeable decrease in *O*-GlcNAc signal (Fig. 5, *a* and *c*). Under the same experimental conditions, treatment with *Ta*OGA54^WT^, but not its inactive counterpart *Ta*OGA54^D121A^, resulted in a dose-dependent decrease in *O*-GlcNAc signal obtained in comparison to untreated controls (Fig. 5, *b* and *d*). To determine specificity of RL-2 to *O*-GlcNAc, lysates were also independently probed with RL-2 antibody preincubated with 0.5 m GlcNAc or secondary antibody alone. The difference in activity observed for *Ta*OGA53 and *Ta*OGA54 on lysates is not unexpected given the kinetic parameters of these enzymes. The ability of *Ta*OGA54 to de-*O*-GlcNAcylate HEK293 and S2 cell lysates demonstrates that *Trichoplax* possesses a functional orthologue of metazoan OGA.

##### Trichoplax Possesses O-GlcNAcylated Proteins

**FIGURE 6.**
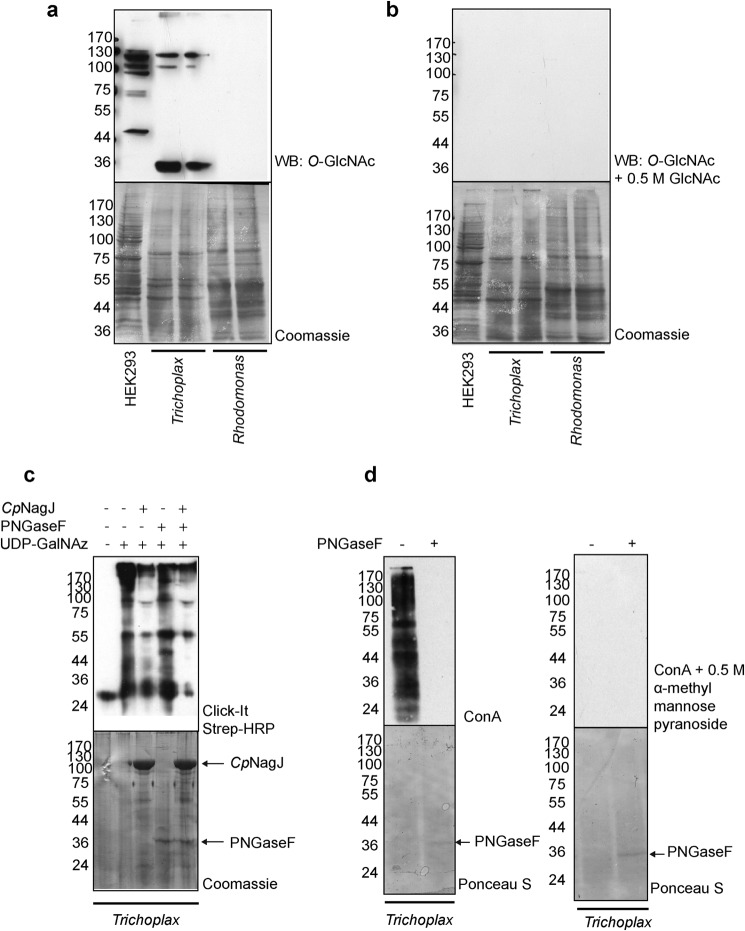
***Trichoplax* possesses *O*-GlcNAc-modified proteins.** *a, Trichoplax* total lysates were subjected to Western blotting using the anti *O*-GlcNAc antibody CTD110.6. HEK293 cell lysates were used as a positive control and *Rhodomonas* lysates were used to ensure the *O*-GlcNAc signal from *Trichoplax* lysates were specific to *Trichoplax* proteins and not contaminating algal proteins in the lysate. *b,* lysates probed with anti-*O*-GlcNAc antibody preincubated with 0.5 m GlcNAc to show specificity of CTD110.6 antibody to GlcNAc. *c, Trichoplax* lysates treated with GalT1^Y289L^ to label *O*-GlcNAc residues with azido-modified galactose (*GalNAz*), which in turn is attached to biotin via click chemistry and probed with peroxidase-conjugated streptavidin (*strep-HRP*) to detect *O*-GlcNAcylated proteins. Lysates were treated with the bacterial *O*-GlcNAcase *Cp*NagJ and/or PNGase F prior to labeling with GalNAz for specific detection of *O*-GlcNAcylated proteins. *d, Trichoplax* lysates subjected to PNGase F treatment were probed with concanavalin A (ConA) to confirm activity of PNGase F on the *N*-glycans of *Trichoplax*. The specificity of ConA was assessed by competing it with 0.5 m α-methyl mannose pyranoside.

Having established that *Trichoplax* expresses functional orthologues of OGT and OGA, we investigated the presence of *O*-GlcNAcylated proteins in the organism by Western blotting using the anti-*O*-GlcNAc antibody CTD110.6. *Trichoplax* lysates were probed for *O*-GlcNAc alongside lysates of *R. salina*, the algal food source used to culture *Trichoplax*, as a negative control. *Trichoplax* lysates showed reactivity toward the antibody, whereas *Rhodomonas* lysates did not, confirming that CTD110.6 reactive proteins were exclusively of *Trichoplax* origin (Fig. 6*a*). Specificity of the signal toward *O*-GlcNAc was determined by preincubating CTD110.6 with 0.5 m GlcNAc, which competed away CTD110.6 reactivity (Fig. 6*b*). The presence of *O*-GlcNAcylated proteins in *Trichoplax* was further confirmed using the alternative “Click-It” approach, whereby *O*-GlcNAc residues are labeled using a mutant galactosyltransferase (Gal-T1^Y289L^) (83) with azido-modified galactose, which is then reacted with biotin-alkyne via copper-dependent cycloaddition and detected by Western blotting using peroxidase-conjugated streptavidin (84). However, the Click-It method could potentially identify any glycosylated protein containing a terminal GlcNAc. *Trichoplax* lysates were therefore treated with PNGase F or *Cp*NagJ, a bacterial OGA (51), to specifically remove *N*-linked glycans or *O*-GlcNAc, respectively, prior to performing the Click-It reactions, to ensure specific detection of *O*-GlcNAc. Although PNGase F treatment did not result in significant reduction in signal obtained with streptavidin-HRP, *Cp*NagJ treatment led to a reduction in signal, establishing the specificity of the results obtained with the Click-It method (Fig. 6*c*). PNGase F-treated lysates were also probed with the lectin concanavalin A (ConA) to ensure *N*-linked glycans were successfully stripped (Fig. 6*d*).

## DISCUSSION

Our data show that the basal metazoan *Trichoplax* expresses functional OGT and OGA and also possesses *O*-GlcNAcylated proteins. Our results suggest that OGT may have a ubiquitous role in *Trichoplax* because its transcripts do not exclusively localize at specific regions of the organism. It is remarkable, given the minimalistic morphology of *Trichoplax*, that *Ta*OGT is able to rescue the lethality of *Drosophila* OGT null mutants. This suggests roles for OGT and protein *O*-GlcNAcylation in evolutionarily conserved processes in *Trichoplax*.

*Trichoplax* is unusual among metazoa in that it encodes two orthologues of OGA. It is possible given the biochemical properties of the shorter *Ta*OGA53 that this is an inactive enzyme with regulatory or scaffolding functions. Although the *K_m_* for *Ta*OGA54 toward the pseudosubstrate 4MU-NAG is about 25-fold higher compared with hOGA (85), its activity on human cell lysates is comparable with that reported for hOGA previously (78), indicating functional conservation of OGA activity throughout metazoan evolution.

The phylogenetic position of *Trichoplax* at the base of metazoa has allowed it to be used in studies investigating the evolution of human cellular pathways and proteins (86, 87). A recent study used comparative genomics to identify metazoan-specific genes defined on the basis of being present in all metazoans including *Trichoplax*, but being absent in other eukaryotes (88). Its presence in *Trichoplax* but not in protists, adds *oga* to this repertoire of metazoan-specific genes. Although the presence of active OGT and *O*-GlcNAcylated proteins in plants and protists (55, 89) implies a eukaryote-specific role for intracellular *O*-GlcNAcylation, it is possible that the post-translational modification is dynamic and reversible only in metazoa. Alternatively, an as yet undiscovered enzyme with low sequence homology to OGA may be responsible for *O*-GlcNAc hydrolysis in protists and plants.

Signal transduction, in part through some types of post-translational modifications of proteins is thought to be one of the prerequisites for multicellularity in metazoa (88, 89). A recent study found higher levels of tyrosine phosphorylation in the proteome of *Trichoplax* than present in more basal or complex organisms, and ascribed it to the appearance of dedicated tyrosine kinases at the onset of metazoan multicellularity (67). Similarly, the acquisition of OGA by *Trichoplax* (and other metazoa) may have enabled the fine-tuning of signal transduction partly via facilitating interplay between *O*-GlcNAcylation and phosphorylation that is known to exist in other organisms (3–5).

We show that *T. adhaerens*, the simplest known animal, is a suitable reductionist model, as it is the most basal organism to possess the machinery required for reversible protein *O*-GlcNAcylation. Despite lacking the genetic tractability of other model systems, *Trichoplax* presents as a useful system to identify conserved *O*-GlcNAc proteins and help shape our understanding of the evolutionary roles for reversible *O*-GlcNAcylation.

## Supplementary Material

Supplemental Data

## Footnotes

2 The abbreviations used are:

OGT: *O*-GlcNAc transferase
OGA: *O*-GlcNAcase
TPR: tetratricopeptide repeat
HAT: histone acetyltransferase
4MU-NAG: 4-methylumbelliferyl-*N*-acetyl-β-d-glucosaminide
PNGase F: peptide *N*-glycosidase F.
