# Supplementary material for: The Early Metazoan Trichoplax adhaerens Possesses a Functional O-GlcNAc System
Source: J Biol Chem. 2015 Mar 16;290(19):11969–82. doi: 10.1074/jbc.M114.628750 (PMC4424335; doi:10.1074/jbc.M114.628750)
Supplement: Supplemental Data [file supp_290_19_11969__index.html]

The early metazoan Trichoplax adhaerens possesses a functional O-GlcNAc system — The Early Metazoan Trichoplax adhaerens Possesses a Functional O-GlcNAc System — O-GlcNAc in the Simplest Known Animal — Supplemental Data 

# The Early Metazoan *Trichoplax adhaerens* Possesses a Functional *O*-GlcNAc System

## Supplemental Data

**Files in this Data Supplement:**

- Supplementary information (.pdf, 11.2 MB) - Supplementary information
