# Supplementary material for: The Early Metazoan Trichoplax adhaerens Possesses a Functional O-GlcNAc System
Source: J Biol Chem. 2015 Mar 16;290(19):11969–82. doi: 10.1074/jbc.M114.628750 (PMC4424335; doi:10.1074/jbc.M114.628750)

The early metazoan *Trichoplax adhaerens* possesses a functional *O*-GlcNAc system

Nithya Selvan[1], Daniel Mariappa[2], Henk W.P. van den Toorn[3], Albert J.R. Heck[3], Andrew T. Ferenbach[1] and Daan M. F. van Aalten[1,2,+]

Supplementary Information

Supplementary Figure Legends

Supplementary Figure S1 – *Trichoplax* possesses an OGT orthologue.

(a) Multiple sequence alignment of *Ta*OGT with *Hs*OGT (hOGT) and *Dm*OGT. Green, purple and pink bars represent the TPR region, the catalytic lobes and the intervening domain respectively of hOGT. The inverted red triangle indicates the key conserved lysine residue involved in catalysis and inverted blue triangles mark the boundaries of the conserved nuclear localization signal.

(b) Surface view of hOGT (PDB: 4AY6) shaded by sequence conservation with *Ta*OGT. Identical residues are in dark blue and similar residues are in light blue, non-conserved residues are in white. The substrate peptide TAB1 (yellow sticks), UDP (cyan sticks) and GlcNAc (magenta sticks) are shown in the active site.

Supplementary Figure S2 - *Trichoplax* possesses OGA orthologues.

(a) Multiple sequence alignment of *Ta*OGA54 and *Ta*OGA53 with hOGA, *Dm*OGA and *Og*OGA. The cyan bar represents the catalytic glycoside hydrolase domain of hOGA, the yellow bar represents the middle domain with the blue line depicting the region of predicted structural disorder in hOGA and the magenta bar shows the HAT- like domain of hOGA. The inverted red triangle shows the conserved catalytic aspartic acid residue of the OGAs and the inverted blue triangle shows the caspase-3 cleavage site in hOGA.

(b) Surface view of *Og*OGA (PDB: 4AY6) coloured by sequence conservation with *Ta*OGA53. Identical residues are in dark blue and similar residues are in light blue, non-conserved residues are in white. The OGA inhibitor PUGNAc (magenta sticks) is shown in the active site.

(c) Surface view of *Og*OGA (PDB: 4AY6) coloured by sequence conservation with *Ta*OGA54. Identical residues are in dark blue and similar residues are in light blue, non-conserved residues are in white. PUGNAc (magenta sticks) is shown in the active site.

Supplementary Figure S1

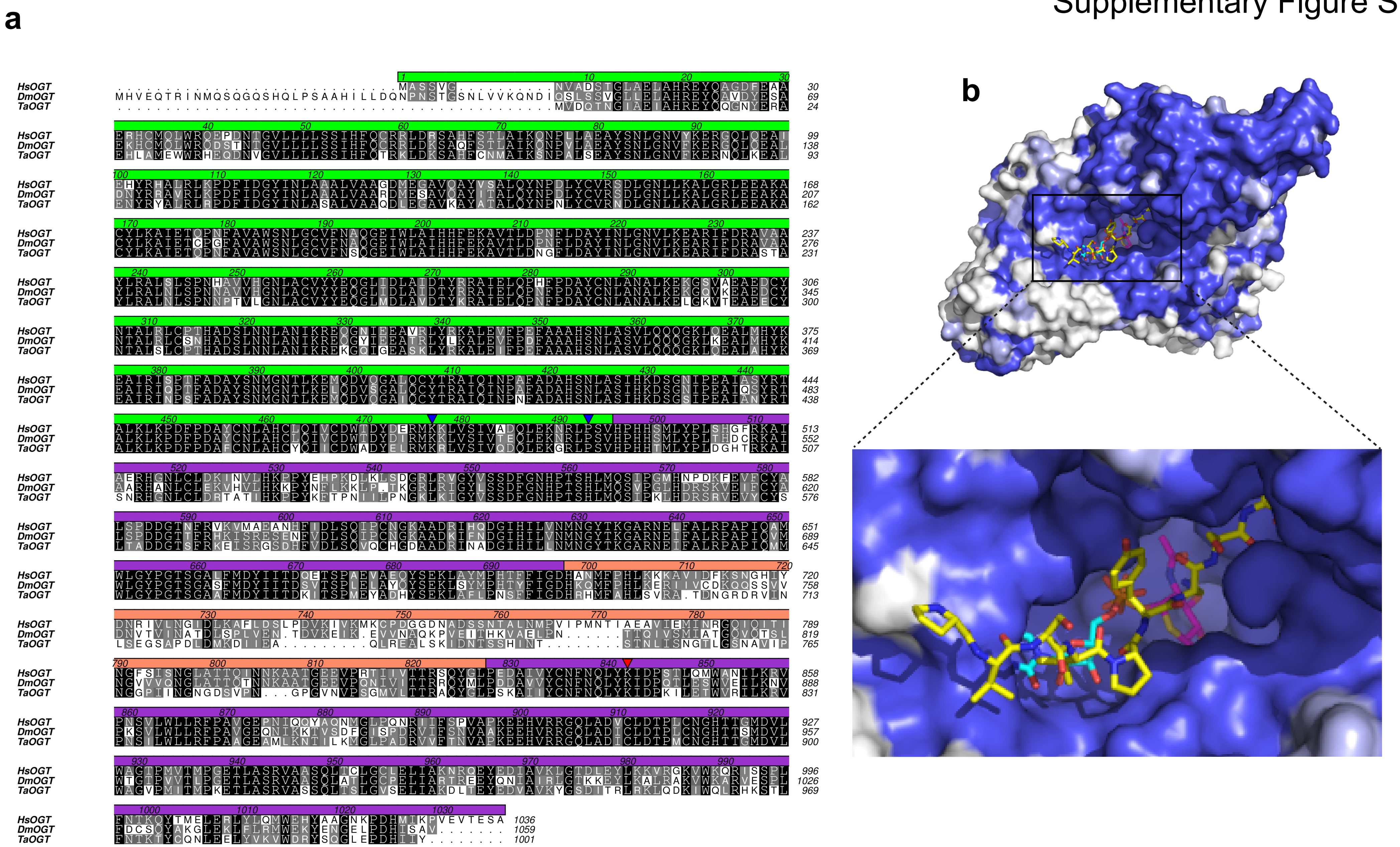

Supplementary Figure S2

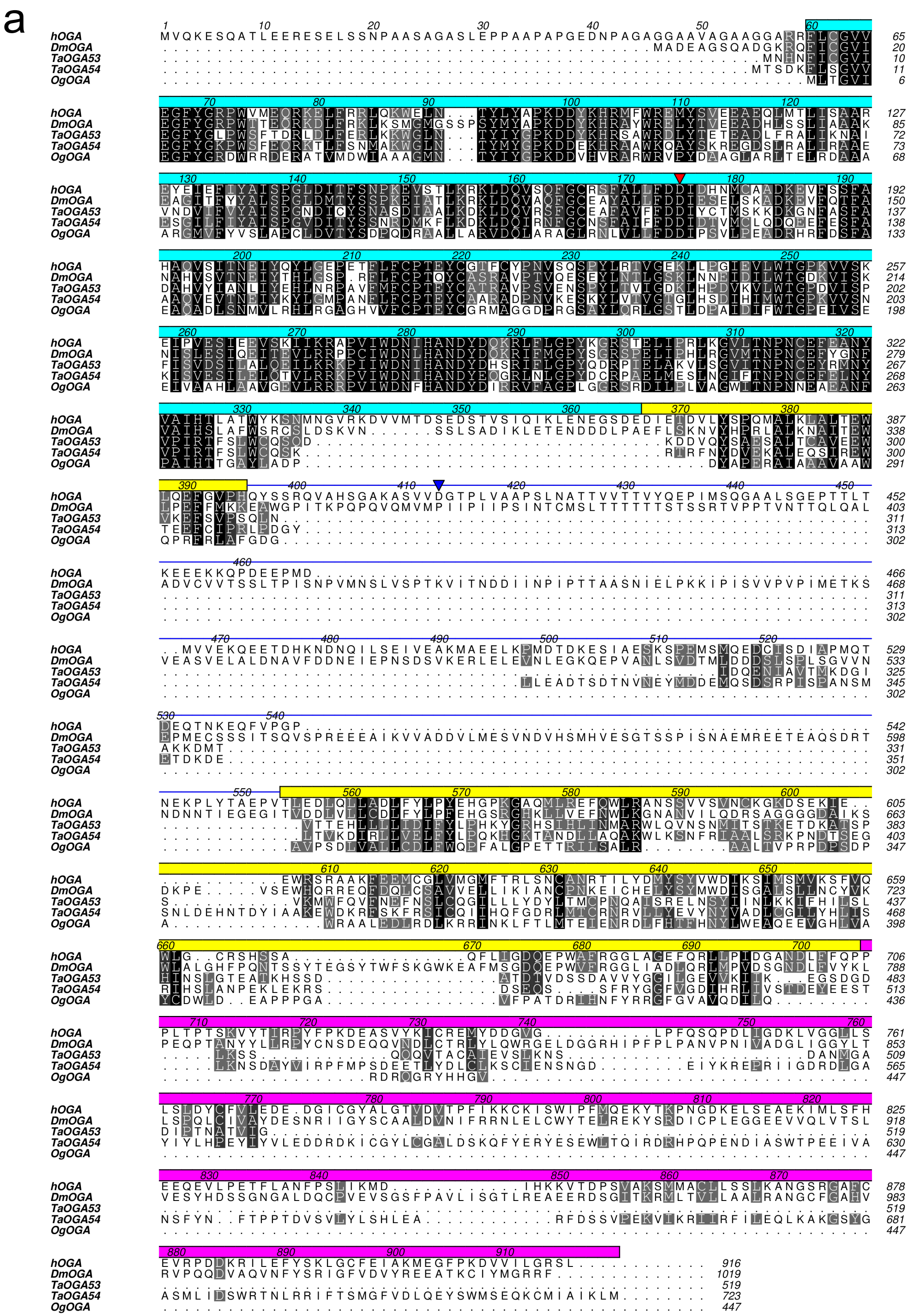

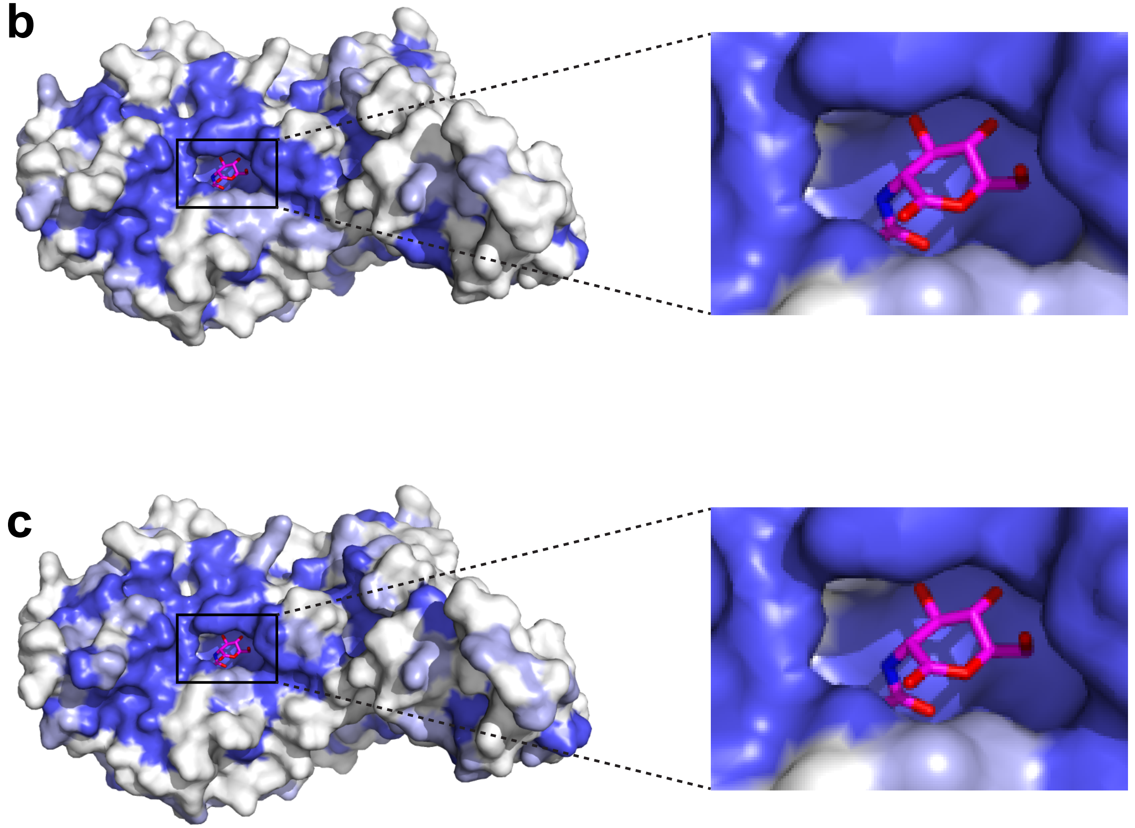

Supplement: Supplemental Data [file supp_M114.628750_jbc.M114.628750-1.pdf]
